# Predictors of psychosocial adaptation and mental wellbeing among people with chronic illness and disabilities in a chinese context

**DOI:** 10.1192/j.eurpsy.2021.2094

**Published:** 2021-08-13

**Authors:** A. Siu, S. Chan, M. Cheung, C. Mo, S. Lai, D. Shek

**Affiliations:** 1 Rehabilitation Sciences, The Hong Kong Polytechnic University, Hunghom, Kowloon, Hong Kong PRC; 2 Rehabilitation Sciences, The Hong Kong Polytechnic Unviersity, Hong Kong, Hong Kong PRC; 3 Rehabilitation Service, Hong Kong Society for Rehabilitation, Hong Kong, Hong Kong PRC; 4 Rehabiltiation Sciences, The Hong Kong Polytechnic Unviersity, Hong Kong, Hong Kong PRC; 5 Applied Social Sciences, The Hong Kong Polytechnic Unviersity, Hong Kong, Hong Kong PRC

**Keywords:** mental well-being, Chronic illness and disability, Psychosocial adaptation, Chinese

## Abstract

**Introduction:**

The process of adjustment to disability and illness among people with chronic illness and disabilities (CID) impacts on motivation for rehabilitation illness self-management, and psychological well-being. It involves a complex interplay of biopsychosocial factors, and was seldom examined in the Chinese context.

**Objectives:**

Identify the predictors of mental well-being of people with from a set of variables including illness and social support, functional abilities, coping strategies, resilience. Examine how these factors interact in determining psychosocial adaptation and mental well-being by structural equations modelling (SEM).

**Methods:**

We conducted a survey of people with CID, who were recruited from community-rehabilitation settings and self-help groups (N = 244). The research questionnaire collected basic demographic information, illness-related variables (e.g. pain, fatigue, functional limitations), and RIDI), social support, measures of resilience, coping, psychosocial ad as predictors, and mental well-being.

**Results:**

Of General Linear Model (GLM) revealed that males have better adaptation than females. Resilience, social coping, & active problem solving are significant predictors of adaptation (Table 1), while age, breathing, pain, resilience, avoidance coping, are predictors of maladaptation (Table 2). A SEM was tested to examine the interaction among the predictors and outcome of adaptation and mental well-being (Figure 1), and the model fit was fair (CFI = 0.89; RMSEA = 0.09)

**Conclusions:**

The results indicated that there are gender differences in adaptation. While all the hypothesized groups of variables contributed to mental well-being of people with CID. Resilience, illness variables, and some forms of coping are closely linked to adaptation and maladaptation.

**Disclosure:**

No significant relationships.

